# Glycosylated Artificial Virus-Like Hybrid Vectors for Advanced Gene Delivery

**DOI:** 10.3390/polym11020243

**Published:** 2019-02-01

**Authors:** Shashank Reddy Pinnapireddy, Mohamed Raafat El Assy, Patrick Schlote, Udo Bakowsky

**Affiliations:** Department of Pharmaceutics and Biopharmaceutics, University of Marburg, Robert-Koch-Str. 4, 35037 Marburg, Germany; amarain_99@hotmail.com (M.R.E.A.); patrickschlote@googlemail.com (P.S.); ubakowsky@aol.com (U.B.)

**Keywords:** polyethylenimine, polyplexes, PEI, liposomes, glycosylation, artificial viral envelopes, gene therapy, nonviral gene delivery

## Abstract

The major obstacle facing efficient gene therapy is the development of reliable delivery vehicles, which are both nontoxic and biocompatible and possess efficient cell-specific gene delivery. Previously, hybrid delivery vehicles comprising anionic liposomes and cationic polymers have been used successfully for gene therapy. In this study, hybrid vectors based on glycosylated artificial viral envelopes (including two novel compositions mimicking HIV and HSV envelopes) and polyethylenimine were morphologically and physiologically characterised. Transfection studies showed that the hybrid vectors based on the control liposomes, and their glycosylated modifications, had significantly higher transfection rates compared to the polyplexes. Improvement in the transfection efficiency was observed with the glycosylated HIV- and HSV-mimicking hybrid vectors, which also showed a safe biocompatibility profile based on the cytotoxicity and haemocompatibility assays. These glycosylated artificial viral envelope-based hybrid vectors could be used as safe gene delivery systems with potential to become new compositions for efficient nonviral gene therapy.

## 1. Introduction

Gene therapy is the therapeutic delivery of DNA, RNA, or antisense sequences into somatic tissue to prevent, modulate, or treat genetic and acquired diseases. This involves genetic diseases caused by a single mutation and acquired diseases for which no single gene mutation causes the disease state. The treatment is targeted at the cause of the disease rather than the symptoms. Designing an effective and safe delivery system, the deficiency of sustained expression, and an immune response are the major challenges involved in gene delivery. For therapeutic activity, a gene delivery vehicle should be biocompatible, protect its cargo from degradation, be nontoxic to the cells, be specific to the target tissue with minimal accumulation in the surrounding tissues and organs, and, most importantly, should not evoke immune responses. Viral vectors, such as retroviruses, adenoviruses, and adeno-associated viral vectors, have already been explored for their therapeutic potential in delivering genetic material. They have a more efficient gene transfer with long-term gene expression than most nonviral vectors, but some clinical trials have shown serious safety problems, such as acute immune responses, immunogenicity, and undesirable mutagenesis as well as restricted trans-gene size [1]. Nonviral vectors on the other hand are cheaper to produce, have a low degree of immunogenicity, and a higher packaging capacity compared to viral vectors [2]. 

Lipids, polymers, and nanoparticles have been used in the recent past for the delivery of genetic material for therapeutic purposes [3,4]. A newer class of hybrid vectors derived from a combination of nonviral vectors have been used successfully in gene therapy, for both in vitro and in vivo studies. These hybrid vectors exploit the merits of liposomes and polymers to create a nanoscale, biocompatible, safe, and efficient delivery system capable of delivering genetic material [5,6]. A blend of anionic liposomes and cationic polymers enables the effective packaging of the genetic material, which protects it from the extracellular environment while at the same time being able to release the cargo intracellularly.

In this study, we formulated and investigated an advanced class of glycosylated lipid-polymer vectors, which mimicked the lipid composition of viral envelopes. The physiological composition of the artificial viral envelopes (AVE) made them serum-resistant and nontoxic. Due to their negative surface charge, different targeting ligands can be attached to the outer surface of the AVEs, such as viral-binding proteins and different sugar moieties [7]. Glycosylation is one of the naturally occurring processes that viruses use to shield the viral protein from immune recognition [8]. Glycosylated carriers, having sugar moieties as surface ligands, are recognised and endocytosed by lectin receptors on the cells [9]. Different types of lectin receptors are expressed on the cell surfaces, such as asialoglycoprotein receptors on parenchymal cells in the liver, mannose receptors on endothelial cells, Kupffer cells in the liver, and lectin receptors on corneal cells in the eye. 

Polyethylenimine (PEI), a cationic polymer known for its ability to efficiently transfect cells, has been employed as the polymer of choice in this study. While the branched variant of PEI is considered relatively cytotoxic, its linear counterpart is less cytotoxic due to the absence of primary amines [10]. Moreover, low molecular weight PEI has a lower toxicity than PEI with a high molecular weight [11]. Although conflicting results are reported in the literature, quite a few studies show that the transfection efficiency of linear PEI is higher than its branched counterpart [6,12].

This aim of this study was to develop hybrid vectors based on glycosylated anionic liposomes that mimic natural viral envelopes (glycosylated artificial viral envelopes (AVE)) for safe and efficient gene therapy. Different hybrid vectors, based on different AVEs, were developed using liposomes that were comprised of viral-mimicking lipids (DPPC, DOPE, DOPS, and cholesterol) and a 22 kDa linear variant of PEI. The hybrid vectors were characterised physicochemically and morphologically using dynamic light scattering, laser Doppler micro-electrophoresis, and atomic force microscopy, respectively. The effect of glycosylation with different glycolipid densities was evaluated as a function of transfection efficiency in vitro using a luciferase reporter assay. The toxicity and haemocompatibility of the hybrid vectors were investigated using MTT and clotting time haemolysis tests, respectively.

## 2. Materials and Methods 

The lipids used for the formulation of liposomes were 1,2-dipalmitoyl-sn-glycero-3-phosphocholine (DPPC), 1,2-dioleoyl-sn-glycero-3-phospho-L-serine (DOPS), and 1,2-dioleoyl-sn-glycero-3-phosphoeth-anolamine (DOPE) and were a kind gift from Lipoid GmbH (Ludwigshafen, Germany). The cholesterol was obtained from Sigma Aldrich (Taufkirchen, Germany). The glycosylated lipid DPPC-galactoside (DPPC-Gal) was synthesised by U. Bakowsky (Martin Luther University of Halle-Wittenberg, Halle, Germany), according to the method of Ogawa [13,14]. A fully deacylated 22 kDa backbone linear PEI with 11% more protonable nitrogen atoms than its 25 kDa counterpart was purchased from Polysciences Europe GmbH (Hirschberg, Germany). The plasmid DNA pCMV-luc used for the luciferase assay was purchased from Plasmid Factory GmbH (Bielefeld, Germany). The SK-OV-3 human ovarian carcinoma cells were procured from the American Type Culture Collection (Manassas, VA, USA) and were cultured in IMDM (Capricorn Scientific, Ebsdorfergrund, Germany) supplemented with 10% foetal bovine serum (Capricorn Scientific, Ebsdorfergrund, Germany) and antibiotics. Cells were grown as monolayers in a humidified incubator with 7% CO_2_ at 37 °C and were passaged upon reaching 80% confluency.

Liposomes were prepared using the thin film hydration technique. For this, the lipids solutions (dissolved in a chloroform and methanol (2:1) mixture) were evaporated in a rotatory evaporator (Laborota 4000, Heidolph Instruments, Schwabach, Germany) to obtain a lipid cake. A total of 20 mM of HEPES buffer (pH 7.4) was added and the mixture was placed in an ultrasound bath above the Tm of the lipids to obtain nanoscaled liposomal vesicles. The liposomes were extruded (Avanti Extruder, Avanti Polar Lipids, Alabaster, AL, USA) through 400 nm and 200 nm polycarbonate membrane filters (Avestin Europe GmbH, Mannheim, Germany) to obtain small unilamellar vesicles of uniform size. Polyplexes of PEI were formed by mixing appropriate amounts (N/P ratio of 10) of PEI and plasmid solutions and were incubated for no more than 30 min at room temperature before being used for further experiments. For lipopolyplex formation, the polyplexes were mixed with the liposomal formulations (polymer–lipid ratio of 2:5) by vigorous pipetting and were incubated for 1 h at room temperature. 

The formulations were characterised for their size (hydrodynamic diameter) and surface charge (zeta potential) using dynamic light scattering (DLS) and laser Doppler micro-electrophoresis techniques on a Zetasizer Nano ZS (Malvern Instruments, Malvern, UK). Morphological analysis was performed by atomic force microscopy (JPK Instruments, Berlin, Germany) using NSC16AlBS scanning probes (Mikromasch, Tallinn, Estonia) in the intermittent contact mode. 

The gene delivery efficiency of the formulations was evaluated using the luciferase reporter assay. Complexes containing 0.1 µg of pCMV-luc plasmid DNA were added to 96-well microtiter plates (SK-OV-3 cells, seeding density 10,000 cells/well). Transfection studies were carried out in a serum-containing medium. After an incubation period of 48 h, the medium was removed and the cells were lysed using a cell culture lysis buffer (Promega GmbH, Mannheim, Germany). Ten mM of D-Luciferin (Synchem UG & Co. KG, Felsberg, Germany) and luciferase assay reagent (Promega GmbH) were mixed together (1:20 ratio). This mixture was injected onto the microplate (50 µl/well) and the luminescence was recorded after 10 s on a plate reader equipped with an auto-injector (FluoStar Optima, BMG Labtech). The corresponding protein content in the lysate was analysed using a Pierce BCA protein assay (Thermo Fisher Scientific GmbH, Dreieich, Germany), in accordance with the manufacturer’s protocol. To determine the cellular uptake pathway of the hybrid vectors, pathway inhibitors inhibiting clathrin-mediated endocytosis (chlorpromazine; Sigma Aldrich) and caveolae-mediated endocytosis (Filipin III; VWR International, Darmstadt, Germany) were used [5]. The inhibition experiments were carried out in an identical manner to the luciferase assay except that the pathway inhibitors were added to the medium (5 µM of chlorpromazine or 3 µM of Filipin III) prior to the transfection. 

Toxicity studies were performed in a similar manner to the luciferase reporter assay with a slight difference that, after 48 h, the medium was removed and replaced with an MTT dye-containing medium. After 4 h incubation, the MTT dye was removed and the formazan crystals resulting from the metabolism of the MTT dye by viable cells were dissolved using DMSO. Absorbance was measured at 570 nm on a plate reader (FluoStar Optima, BMG Labtech) with the absorbance of untreated cells (blanks) being considered as 100% viability.

Haemocompatibility studies to determine the biocompatibility of the hybrid vectors for intravenous administration were performed using fresh blood from healthy donors drawn into citrate tubes, with their prior consent. The blood was centrifuged briefly to separate the plasma fraction from the erythrocytes which were subsequently washed with and diluted (1:50) with 0.9% NaCl solution [15]. Complexes containing 1 µg of plasmid DNA were incubated with the erythrocytes (1:1 volume ratio) for 1 h at 37 °C in a shaking incubator (IKA KS 3000, IKA Werke, Staufen, Germany). The samples were centrifuged, and the supernatants were analysed spectrophotometrically at 570 nm (FluoStar Optima, BMG Labtech, Offenburg, Germany). For the clotting time analysis (activated partial thromboplastin time (aPTT)), the blood plasma was mixed with complexes containing 1 µg of plasmid DNA. The samples were placed in a coagulometer (Coatron M1, Teco GmbH, Neufahrn, Germany), and the clotting time was determined using a TEClot aPTT-S kit (Teco GmbH) by following the manufacturer’s protocol.

All experiments were performed in triplicate and the results are reported as averages of these measurements ± standard deviation. A two-tailed student’s t-test was performed to determine the significance of differences between the measurements. The probability values of *p* ≤ 0.05 were considered significant. Significance levels are denoted as * for *p* ≤ 0.05, ** for *p* ≤ 0.01, and *** for *p* ≤ 0.001.

## 3. Results and Discussion

With the aim of investigating the effect of glycosylation on gene transfer efficiency, liposomal formulations (both native and glycosylated) aimed at mimicking the HIV and HSV viral envelopes were prepared. Using a mixture of DOPE, DPPC (or DPPC-Gly), and DOPS, which are found in these viral envelopes [16,17], liposomes (abbreviated as AVE for artificial viral envelope or gAVE for glycosylated artificial viral envelope) were prepared with varying molar ratios, as indicated in Table 1. Cholesterol was added to the liposomal formulations to enhance the mechanical stability of the lipid membranes [18]. The hydrodynamic diameter and zeta potential measurements were measured on a Zetasizer, and the values of the three independent formulations are denoted below. AVE1 and AVE2 formulations mimic HIV and HSV viral lipid compositions, respectively.

Since the artificial viral-like liposomes were ultimately meant to encapsulate the polyplexes, which were around 100 nm in size (Table 2), extrusion was done using 400 nm and 200 nm polycarbonate membranes, respectively, to yield uniform homogenous vesicles. All the liposomal formulations were below 130 nm and were monodisperse (Polydispersity index (PDI) below 0.1) with zeta potentials ranging from 25 to 40 mV. After initial studies involving different N/P ratios of the polyplexes and the mass ratios of polyplexes to liposomes, further experiments were narrowed down to an N/P ratio of 10 for the polyplex formation and a mass ratio of 2:5 (polyplex to liposome) for the hybrid vector formation (abbreviated as HV, or gHV for hybrid vectors formulated using glycosylated liposomes). A complex size of 200 nm and less is the desirable size for endocytosis and efficient cellular uptake [19]. Considering this number, formulations exceeding this size range were excluded from further studies. 

As the values of the physicochemical characterisation indicate, the hybrid vectors (formed using both native liposomes and their glycosylated counterparts) were the desirable size of <200 nm. The polydispersity index, in the case of the hybrid vectors, was slightly higher due to the presence of liposomes and polyplexes as a single entity. The net positive zeta potential could be attributed to the radiation of the positive charge of PEI to the outside of the liposomal bilayer, which is usually 4 nm in thickness [20].

**Figure 1:**
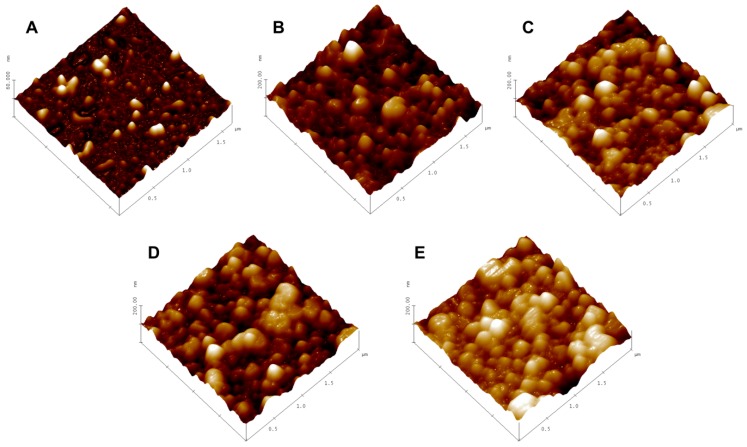
(**A**) AFM micrograph of linear polyethylenimine (PEI) polyplexes; (**B**–**E**) AFM micrographs of HV1, gHV1, HV2, and gHV2 hybrid vectors, respectively. Images were obtained in the intermittent contact mode at a resonance frequency of 170 kHz and a force constant of 40 N/m. Scale bars across all the micrographs represent 2 µm.

Atomic force microscopy studies were carried out, after letting the samples settle on a mica chip fixed on a glass slide. The sample excess was shaken off after a few minutes and the sample was allowed to air dry. The samples were visualised under the intermittent contact (tapping) mode to minimise any damage to the samples while acquiring an image. The scan rates of 0.5 to 1 Hz were used to acquire images of the polyplexes and hybrid vectors. The AFM micrographs (Figure 1) of the hybrid vectors (Figure 1B–E) show spherical-shaped vesicles within the size range obtained by the DLS measurements measured using a Zetasizer. The hybrid vectors appeared to be aggregated together, which is due to the fact that the vesicles tended to move closer on the mica chip during the preparation and air-drying process. The micrograph of the polyplexes (Figure 1A) shows irregularly shaped complexes corresponding with the size determined by the DLS.

**Figure 2:**
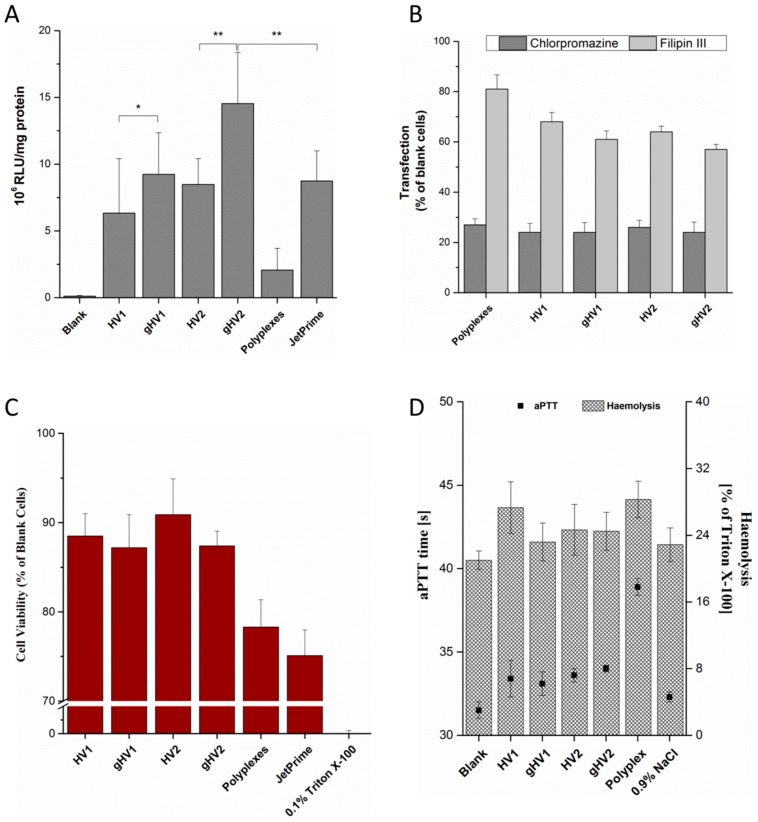
(**A**) Transfection efficiency (luciferase reporter assay) of the hybrid vectors in a SK-OV-3 cell line. Blank denotes untreated cells. Data are expressed in relative luminescence units per mg protein (evaluated using a Pierce BCA assay). JetPrime, a commercially available polymer-based transfection reagent, was used a control for the transfection studies; (**B**) uptake pathway inhibition of the hybrid vectors in the presence of either 5 µM of chlorpromazine or 3 µM of Filipin III in a SK-OV-3 cell line. Blank cells (y-axis) denote the transfected cells without pathway inhibitors. Values are denoted as percentages of the luciferase expression of cells transfected with their respective vectors (either polyplexes or hybrid vectors); (**C**) MTT assay of the hybrid vectors in a SK-OV-3 cell line. Cell viability is expressed as the percentage of blank cell viability, with 0.1% Triton X-100 as a positive control; (**D**) haemolysis and activated partial thromboplastin time test of the hybrid vectors using erythrocytes and blood plasma, respectively, with 0.9% NaCl as a control. Blank denotes untreated erythrocytes in the case of haemolysis and untreated plasma in the case of the aPTT test. HV1 and HV2 denote the hybrid vectors with lipid composition mimicking that of HIV and HSV, respectively, and gHV1 and gHV2 denote their glycosylated counterparts.

A luciferase reporter gene assay was performed to assess the in vitro efficiency of the hybrid vectors. The hybrid vectors formulated using the PEI polyplexes were incubated with the cells for 48 h before being evaluated. The luciferase activity resulting from the transfection of the cells with the pCMV-luc plasmid is directly proportional to the transfection efficiency of the vectors. Compared to polyplexes, the hybrid vectors showed a tremendous increase in the transfection efficiency with more than a five-fold increase compared to gHV2 hybrid vectors (Figure 2A). As expected, the transfection efficiency of glycosylated artificial viral vectors was significantly greater than their native counterparts, suggesting a more efficient cellular internalisation— a characteristic of viruses [21]. The transfection efficiency of the gHV2 hybrid vectors was significantly higher than the commercially available polymer-based transfection reagent, JetPrime (Polyplus-transfection SA, Illkirch, France). Interestingly, the HSV virus-mimicking hybrid vectors were more efficient than their HIV-mimicking counterparts. The reason behind this behaviour is, however, not clear since the uptake mechanisms of all the hybrid vehicles were relatively similar (Figure 2A). Uptake studies were designed to understand the exact mechanism lying behind the internalisation of the hybrid vectors. Chlorpromazine and Filipin III were used as inhibitors of clathrin- and caveolae-mediated endocytoses, respectively. The results of the inhibition studies show a sharp decrease in the transfection efficiency in the presence of chlorpromazine for both the polyplexes and the hybrid vectors, suggesting a clathrin-mediated endocytosis pathway. However, in the case of the inhibition of the caveolae-mediated endocytosis, it appeared that the hybrid vectors had, to some extent, been inhibited in the presence of Filipin III. There was also a slight difference in the inhibition levels between glycosylated and nonglycosylated hybrid vectors, which correspond with the results of the luciferase assay—a similar trend in luciferase expression. This could possibly be due to the ability of the glycosylated hybrid vectors to use both forms of endocytosis to a certain extent [22]. This also correlates with studies confirming the positive effects of glycosylation on cellular uptake [23,24]. 

Toxicity studies were performed to determine the biocompatibility of the hybrid vectors. After the transfection of cells with polyplexes and hybrid vectors containing pCMV-luc plasmid DNA, the MTT dye was added to determine the percentage of viable cells that were capable of metabolising and converting the MTT dye into DMSO-soluble formazan crystals. The viability of the hybrid vectors was found to be above 85% (Figure 2C). Linear PEI polyplexes also showed a viability of nearly 80%, which was higher than the polymer-based transfection reagent, JetPrime. The increase in the cell viability of the hybrid vectors is due to the presence of the lipid layer, which helps shield the excess positive charge radiating from the PEI polyplexes, as suggested previously [6]. It is worth mentioning that both the polyplexes and hybrid vectors were incubated with the cells for 48 h without any change in the medium during the experiment. To further understand the biocompatibility of the hybrid vectors and to determine their suitability of intravenous application, haemocompatibility tests were performed. These studies involved testing the compatibility of the hybrid vectors with erythrocytes and determining their effect on blood coagulation. A haemolysis test was performed with the erythrocytes isolated from fresh blood, indicating an increase in the haemolytic potential by 2–10% compared to the untreated erythrocytes (Figure 2D). Upon possible rupture, erythrocytes release haemoglobin which reacts with atmospheric oxygen to form oxy-haemoglobin, which can be quantified spectrophotometrically. Under physiological conditions, however, upon intravenous administration, the formulations quickly get distributed in the blood stream with minimal interaction with individual blood components. It should, however, be taken into consideration that high haemolysis values could, irrespective of the interaction time, show deleterious effects. Another important aspect in determining biocompatibility, with respect to intravenous administrations, is the effect on blood coagulation time. The activated partial thromboplastin time helps determine the safety profile of the formulation in terms of a possible increase in the coagulation time [25]. The hybrid vectors used in this study showed only a marginal increase in the clotting time (by 2–3 s compared to the blanks). In the case of polyplexes, this increase was more pronounced (by 6 s compared to the blanks). Haemocompatibility studies serve to bridge the gap between in vitro and in vivo studies and help determine the safe concentration for in vivo studies.

## 4. Conclusions

The artificial virus-like nanoscaled hybrid delivery vectors designed using PEI polyplexes and viral-mimicking liposomes showed an improved transfection efficiency compared to PEI-based polyplexes. The glycosylated versions of these hybrid vectors showed further improvement in the transfection efficiency, which could be attributed to their dual internalisation pathways. These hybrid vectors showed a safe toxicity and haemocompatibility profile. This is a prerequisite for in vivo studies, which would be our prime research interest with the goal of making these hybrid vectors potential contenders for safe and efficient gene therapy.

## Figures and Tables

**Table 1:** Hydrodynamic diameter (by intensity) and zeta potential of the liposomal formulations.

Liposomes (Mol%)	Size (nm)	Zeta Potential (mv)	PDI
DOPE:DPPC:DOPS:Cholesterol (AVE1) (25:30:15:30)	119.6 ± 3.1	−29.2 ± 4.4	0.10
DOPE:DPPC-Gal:DOPS:Cholesterol (gAVE1) (25:30:15:30)	127.3 ± 2.8	−25.4 ± 2.0	0.08
DOPE:DPPC:DOPS:Cholesterol (AVE2) (30:50:5:15)	123.1 ± 7.4	−38.5 ± 4.2	0.11
DOPE:DPPC-Gal:DOPS:Cholesterol (gAVE2) (30:50:5:15)	128.9 ± 5.0	−33.1 ± 4.7	0.10

All values are indicated as the mean of three independent (n = 3) measurements ± SD.

**Table 2:** Hydrodynamic diameter (by intensity) and zeta potential of the liposomal formulations.

Complexes	Size (nm)	Zeta Potential (mv)	PDI
Linear PEI-pCMV-luc polyplexes	96.2 ± 11.3	+19.2 ± 3.1	0.18
AVE1 hybrid vectors (HV1)	181.3 ± 9.7	+8.2 ± 1.8	0.20
gAVE1 hybrid vectors (gHV1)	194.8 ± 12.5	+15.1 ± 2.6	0.27
AVE2 hybrid vectors (HV2)	188.6 ± 10.8	+11.8 ± 5.1	0.22
gAVE2 hybrid vectors (gHV2)	185 ± 14.9	+13.8 ± 3.5	0.21

All values are indicated as the mean of three independent (n=3) measurements ± SD.
